# Challenges in managing osteogenesis imperfecta patients in H. Adam Malik hospital, North Sumatera, Indonesia

**DOI:** 10.1186/1687-9856-2013-S1-P162

**Published:** 2013-10-03

**Authors:** Siska Mayasari Lubis, Melda Deliana, Hakimi

**Affiliations:** 1 Pediatric Endocrinology Division, Child Health Department, Medical School, University of Sumatera Utara, H.Adam Malik Hospital, Medan, Indonesia

## Background

Osteogenesis imperfecta (OI) is a genetic disorder with increased bone fragility and low bone mass, the incidence has been estimated at 1 in per 10000-20000 births, however milder forms of OI are probably under recognized. Cyclical intravenous therapy with the biphosphonate pamidronate has been reported to be beneficial in children and adolescents with moderate to severe forms of OI.

## Objective

To report the challenges in managing osteogenesis imperfecta patients in H. Adam Malik Hospital, a referral hospital in North Sumatera, Indonesia.

## Case

From July 2011 until now, we have 5 cases of OI in our hospital; all of them were type 3. The youngest was 3 days old and the oldest was 9 years old, all of them were male. These patients have well defined phenotypes, including extremely short stature, progressive limb and spine deformities. We found respiratory problems in 2 patients, a 3 days and 2 months old baby. Diagnosis was based on clinical and radiology examination (bone survey). All of them came from low social economic status, and only 2 patients had government health insurance. The major problem in managing OI patients is the treatment, intravenous pamidronate was not available in our province therefore we must order it from our national referral hospital in Jakarta. Moreover, the price was expensive, and it was not covered by health insurance. Only 1 patient received this treatment and only for 1 cycle. One patient died at home because of respiratory problem. BMD or DXA was also not available in our province.

## Conclusion

Managing OI patients in our hospital is still be a big challenge especially regarding the treatment, needing collaboration from all the part concerning the patients, including attention and support from the government, community, and medical provider.

